# A New Internet of Things Hybrid VLC/RF System for m-Health in an Underground Mining Industry

**DOI:** 10.3390/s24010031

**Published:** 2023-12-20

**Authors:** Daniel Iturralde, Javier Guaña-Moya, Pablo Palacios Játiva, Iván Sánchez, Muhammad Ijaz, Ali Dehghan Firoozabadi, David Zabala-Blanco

**Affiliations:** 1 Department of Electronic Engineering, Universidad del Azuay, Cuenca 010204, Ecuador; diturralde@uazuay.edu.ec; 2 Facultad de Ingeniería, Pontificia Universidad Católica del Ecuador, Quito 17012184, Ecuador; 3 Escuela de Informática y Telecomunicaciones, Universidad Diego Portales, Santiago 8370191, Chile; pablo.palacios@mail.udp.cl; 4 Department of Telecommunication Engineering, Universidad de Las Américas, Quito 170503, Ecuador; 5 School of Engineering, Manchester Metropolitan University, Manchester M15 6BH, UK; m.ijaz@mmu.ac.uk; 6 Department of Electricity, Universidad Tecnológica Metropolitana, Santiago 7800002, Chile; adehghanfirouzabadi@utem.cl; 7 Department of Computing and Industries, Universidad Católica del Maule, Talca 3480112, Chile; dzabala@ucm.cl

**Keywords:** IIoT, m-health, mining

## Abstract

This paper proposes a new system based on the Industrial Internet of Things (IIoT) for the monitoring of Mobile Health (m-Health) of workers in the underground mining industry. The proposed architecture uses a hybrid model in data transmission. Visible Light Communication (VLC) is used for downlink because of its narrow coverage, which aids in worker positioning. Radio frequency (RF) communication technology is used to send data for primary vital signs in the uplink, which is more efficient in transmission and is a viable solution according to the problem raised. The results obtained in terms of coverage and transmission for the downlink and uplink links show the feasibility of implementing the proposed system.

## 1. Introduction

The International Labor Organization (ILO) states on its website that every 15 s, 153 workers have an accident at work, and annually more than 317 million accidents occur at work, many of which result in absenteeism. In the same way, ILO affirms that every 15 s a worker dies from accidents or work-related illnesses, that is, every day 6300 people die from that cause, equivalent to more than 2.3 million deaths per year. There are work environments that are, by nature, more dangerous than others. This is the case for the mining industry, where 12,000 workers die every year in mining accidents around the world [1].

Accident rates within the mining industry persist at elevated levels, underscoring the need for a stringent legal framework that requires employers to proactively institute comprehensive measures aimed at preventing workplace setbacks. Recognizing the urgency of addressing this issue, the United States Congress took decisive action in summer 2006. Responding to a poignant series of tragedies that occurred earlier in that year, the legislative body enacted the Mine Improvement and New Emergency Response (MINER) law [2].

The MINER law is a crucial piece of legislation that places a compelling obligation on mine managers not only to implement safety protocols, but also to submit a meticulously designed emergency response plan. This multifaceted strategy seeks to mitigate the occurrence of accidents while ensuring a rapid and effective response in the unfortunate event of an emergency [3]. The emphasis of the law on proactive planning and preparation reflects a paradigm shift towards a more comprehensive and preventive approach to occupational safety within the mining sector. Consequently, it serves as a critical milestone in ongoing efforts to improve workplace safety standards and protect the well-being of those employed in this highly risky industry [3].

In this sense, this work proposes a new Internet of Things system to monitor the health of mobile workers in the underground mining industry. The Wireless Body Area Network (WBAN) placed in the mining workers uses an optical receiver that obtains a cell ID sent by the infrastructure through a Visible Light Communication (VLC) link based on optical cells, which is used for joint positioning and communication within the mining tunnel and Radio Frequency (RF) for the transmission of primary vital signs to the cloud. Once the data are obtained, a medical professional located outside the mine can observe and control the previously processed data.

The manuscript has the following organization. Section 2 presents a review of wireless communication technologies for m-Health, Section 3 presents the state of the art in m-Health technologies, Section 4 establishes the network architecture, and Section 5 shows the simulation results. Finally, Section 6 is dedicated to conclusions based on the results obtained.

## 2. Wireless Communications Technologies for m-Health

Mobile Health (m-Health) is broadly defined as a medical practice supported by mobile devices, such as mobile phones, health monitoring devices, Personal Digital Assistants (PDAs), and other wireless devices. Connectivity plays an important role in patient monitoring, and it has become particularly critical in those applications where it is necessary to move patient data to data centers. A wide range of innovative wireless technologies, such as Bluetooth Low Energy (BLE), Infrared (IR), Wireless Local Area Network (WLAN), ZigBee, and VLC are increasingly being used in medical devices, especially those used for patient monitoring applications [4].

**Figure 1:**
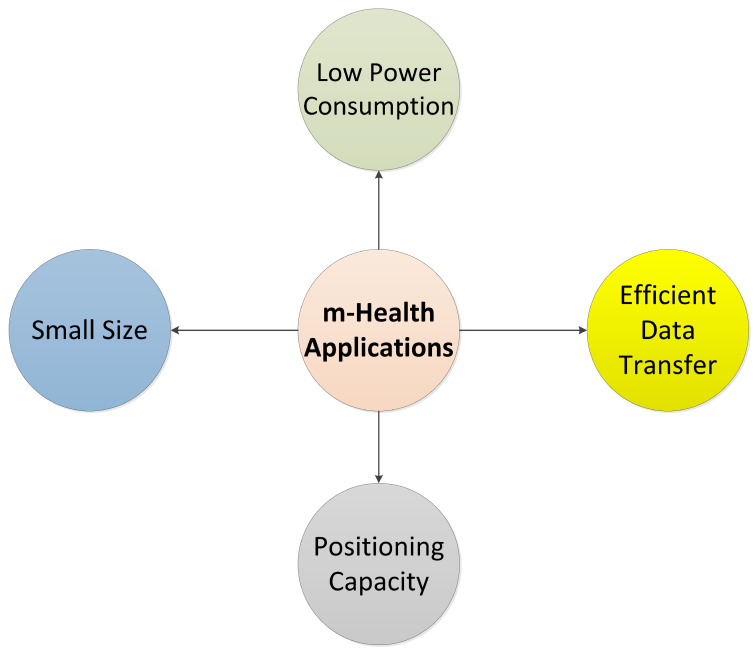
m-Health Requirements.

As shown in Figure 1, the essential requirements for m-Health monitoring devices are small size, low power consumption, positioning capacity, and the ability to efficiently and accurately transfer data to remote monitoring stations [5].

### 2.1. RF Based Technologies

- **Bluetooth:** ref. [6] The Bluetooth Low Energy (LE) radio is designed to operate at very low power levels, from 1 mW to 100 mW, operates in the 2.4 GHz frequency band, and supports data rates of 125 Kbps up to 2 Mbps. The coverage distance can be 100 m [7].
- **Wi-Fi:** ref. [8] Wi-Fi is a family of wireless network protocols based on the IEEE 802.11 [8] family of standards, which are commonly used for local area networks of devices and Internet access. Wi-Fi works in frequency bands of 2.4/5 GHz and supports data rates from 1 Mbps to 1 Gbps. The maximum coverage distance can be 100 m [9].
- **Zigbee:** ref. [10] Zigbee is a specification based on IEEE 802.15.4 [10] for a set of high-level communication protocols used to create personal area networks. It operates in the 2.4 GHz frequency band, supports data rates of 250 kbps, and reaches coverage ranges of up to 300 m [11].

### 2.2. Optical Wireless Communications

- **IR:** IrDA provides specifications for a complete set of protocols for wireless infrared communications. The main characteristics of this kind of wireless optical communication are physically secure data transfer, line-of-sight (LOS), and a very low bit error rate (BER) that makes it very efficient. Works in the frequency band of 100 to 200 THz, has data rates supported at 1 Gbps and a maximum range of 5 m [12].
- **VLC:** [13] The operation of Li-Fi is quite simple, since the data stream is sent by an LED bulb for transmission, which is simply embedded in the beam of light when we see it illuminate the surrounding environment. The 0s and 1s representing the information being sent are presented by the very fast dimming of the LED bulb. The use of visible light means that the Li-Fi signal does not pass through walls, so it is only suitable for display within a room [14,15,16,17,18].

### 2.3. Comparison of Wireless Communication Technologies

Table 1 gives some distinguishing features and important comparisons between OWC and RF technologies. OWC compared to RF technologies does not constitute or suffer from electromagnetic interference and offers a huge unregulated bandwidth. OWC is relatively safe from potential hackers, as the optical radiation is very well confined and does not penetrate walls, compared to RF, where the data security is poor. This advantage present in VLC is in turn a disadvantage from the point of view of mobility and coverage, as RF has a wide coverage and therefore allows mobility for users.

## 3. State of Art

Some of the papers that deal with this topic are discussed in the following.

In [19], an implementation of mobile VLC technology is presented for the transmission of clinical data in the m-Health system at home is presented; multiple clinical data such as an Photoplethysmogram (PPG), Electrocardiogram (ECG), and breathing signals are transmitted through Light Emitting Diodes (LEDs) and received by the camera of a smartphone. In [20], optical modulation based on the hospital lighting network is used; high-brightness LED (HB-LED) is used in this system as a source of lighting so that general hospital information is modulated in the visible light emitted. In [21], a new VLC technology is proposed to transmit and monitor biomedical data; white-beam LEDs are used as optical information emitters to efficiently transmit biomedical data; On-Off Keying (OOK) modulation is used to modulate data in the visible light beam, and a high-speed photodetector (PD) is used in the receiver. In [22] there is a static design for monitoring patients indoors using VLC as an appropriate uplink for hospital settings; the proposed system is designed to transmit uplink patient data using VLC through OOK modulation. In [23] a non-invasive Electroencephalogram (EEG) monitoring system based on visible light communication is deployed using a generic microcontroller. In [24] a method that uses VLC technology as a transmission platform and provides real-time monitoring of heart rate, and patient information is analyzed; the white LED beam is used as a light source to simultaneously transmit the biomedical signal and the patient’s medical record; the OOK modulation technique is used to modulate all data in the visible light beam. At the other end, a receiving circuit consisting of a high-speed photodetector and a demodulation circuit is used to demodulate the visible light beam data; demodulated data are transmitted in series to a personal computer where the biomedical signal, patient information, and heart rate can be monitored in real time. In [25,26] a new strategy for the transmission of the biomedical signal of the EEG is described using a VLC link; data transmission is performed under the Line of Sight (LOS) condition using OOK modulation and the three LED color components: Red, Green, and Blue (RGB). In [27], the focus is on the optical transmission of EEG signals that use an LED and a PD to display the information in the LabVIEW 2022 software environment.

In [22], a new Optical Body Area Network (OBAN) is presented to efficiently transmit multiple human vital signs of patients. The main human vital signs are body temperature, pulse rate, respiratory rate, and blood pressure. These vital signs are captured from four different patients and transmitted by VLC. Multi-patient OBAN uses orthogonal codes to transmit the data of each patient through VLC, and the OOK modulation technique is used to transmit the coded human vital signs of multiple patients. In [28], a Time-Hopping (TH) VLC system is proposed; the TH scheme is adopted to transmit multiple medical data while maintaining a simple system design. In [29], Sparse Code Multiple Access (SCMA) is used to transfer multiple medical data with greater bandwidth efficiency in real-time. The received signal, which includes multi-user data, is separated by the message-passing algorithm with high reliability and is simultaneously reorganized for transmission to a user interface.

In [30,31,32], VLC system designs for portable patient monitoring devices are presented; it is a two-way communication system, where downlink communication uses VLC, while uplink communication uses infrared. In [33] an integrated system is proposed through Power Line Communications (PLC) and VLC with Orthogonal Frequency Division Modulation (OFDM) for indoor hospital applications. In [34] a hybrid system of RF communication and VLC with double-hop is modeled to provide a solution to communicate the results of laboratory tests to patients in a short time.

As has been shown, e-Health research in OWC systems has mainly focused on unidirectional biosignal transmission systems from a single user, unidirectional systems from multiple users, and hybrid transmission systems that allow bidirectional biosignal communication. In this context, it is important to consider that all these works have been developed in hospitals or common indoor environments where the systems are static and mobility is not necessary.

In the application scenario described in this article, a VLC link is used for the downlink, while an RF link is used for the uplink. This approach offers several advantages compared to previous methods. The use of VLC for downlink communication provides high positioning precision, and unlicensed operation, and does not present radio frequency-based interference, making it suitable for indoor environments. Furthermore, the VLC system does not pass through walls, providing a level of security against potential hackers and electromagnetic interference, which is not the case with RF technologies [15,16,17,18].

On the other hand, RF technology is used for uplink communication due to its wide coverage and mobility support, making it suitable for transmitting data from the monitoring center to the network and the Internet. This combination of VLC for the downlink and RF for the uplink leverages the strengths of each technology to optimize communication in the given application scenario. Therefore, the use of VLC for downlink and RF for uplink in this scenario provides a balance between precision, security, and coverage, which is advantageous compared to previous methods that may have relied solely on RF or other wireless communication technologies.

## 4. Network Architecture

**Figure 2:**
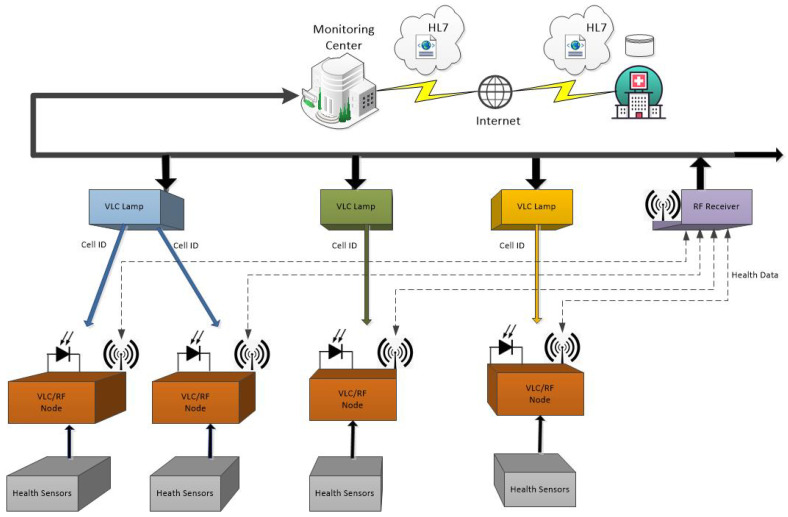
Network architecture.

As shown in Figure 2, an IoT approach is provided by connecting multiple wearable sensors placed in miners that connect to the Internet. These wearable sensors collect and share primary vital sign data for use and evaluation by health-related organizations. Wearable sensors must be connected to a network so that the collected data can be saved and shared. For this purpose, VLC lamps and RF receivers are used. VLC lamps that use different frequencies are responsible for sending an identifier for the correct positioning of the miner, collecting the health data of the miners, and providing connectivity to the network and the Internet, an uplink based on RF technology is used. The monitoring center may have the ability to make immediate decisions or send the data over the Internet for further analysis to storage in a database using a standard recognized as HL7 [35].

### 4.1. VLC Downlink

With the concept of intelligent environments and the Internet of Things (IoT), we face a high level of activity in indoor positioning systems. The RF-based Global Positioning System (GPS) is one of the most reliable positioning techniques, which has been widely used in outdoor environments. However, GPS-based systems have problems in indoor environments concerning low positioning accuracy. To complement GPS for indoor applications, several wireless positioning technologies occupy RF such as RFID, Wi-Fi, ZigBee, and Bluetooth, and these technologies offer low positioning accuracy (PA) due to multipath. Instead, VLC offers several advantages, including high positioning precision; and unlicensed operation; moreover, it is not harmful to human health and does not present radio frequency-based interference [14].

**Figure 3:**
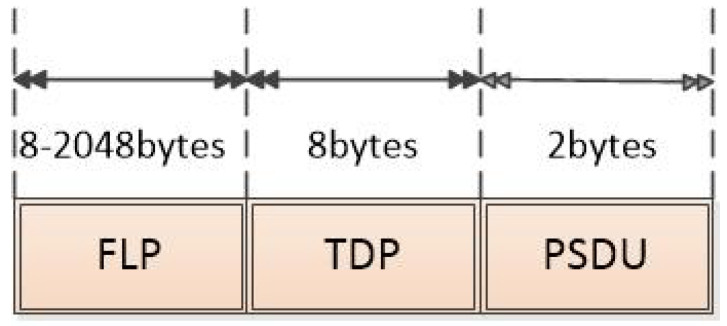
VLC Frame.

The IEEE 802.15.7 standard for Personal Area Networks in Wireless Optical Communications (OWPAN) relates the intended application with the broadcast topology, and the infrastructure will be able to transmit the cell identifier to the mobile nodes without forming a network with them, in a unidirectional communication and for which the destination address is not required. The standard defines the PHY II physical layer mode used mainly for indoor environments that allows one to use an OOK modulation with Reed Solomon Forward Error Correction (FEC) encoding. The physical layer supports a single data transmission mode that transfers one Physical Protocol Data Unit (PPDU) per frame, the structure of the PPDU frame is formatted as illustrated in Figure 3 [13].

The transceiver uses the preamble field to obtain synchronization of the optical clock with an incoming message. The standard defines a Fast Blocking Pattern (FLP) followed by a selection of four Topology-Dependent Patterns (TDPs) to distinguish different PHY topologies. The preamble will be sent at a clock rate chosen by the Transmitter (TX) and supported by the Receiver (RX). The preamble is a sequence in the time domain and has no channel encoding or line encoding. The PSDU field has a variable length and carries the PHY frame data.

**Figure 4:**
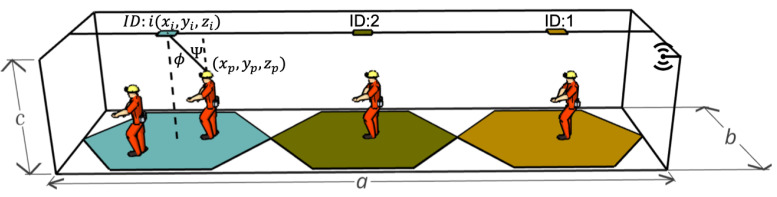
VLC Downlink.

A tunnel space is considered with dimensions *a* m × *b* m × *c* m, similar to Figure 4; this space is divided into several hexagonal optical cells. In each of these cells, there is an LED *i* that transmits a cell identifier on the air and provides coverage to the region limited by that cell at a specific frequency, which can be reused in a different cell with a minimum distance from the first, thus avoiding adjacent channel interference.

To generate the downlink communication link, VLC transmitters using an LED *i*, which is placed on the tunnel roof at coordinates $\left(x_{i},y_{i},z_{i}\right),\forall i\in \left(1,2,\ldots ,i\right)$ and *p* mobile transceivers containing a photodetector and an RF emitter are placed on the safety helmet of mining personnel with coordinates $\left(x_{p},y_{p},z_{p}\right),\forall p\in \left(1,2,\ldots ,p\right)$, in addition to Ψ which is the angle of incidence of light and where ϕ is the angle of irradiation.

The optical power received by each photodetector *p* from the LED *i* is described as:

(1) $$Pr_{\left(i,p\right)}=Pt_{i}Hlos_{\left(i,p\right)},$$

where, $Pt_{i}$ is the power transmitted by LED *i*. The channel response $Hlos_{\left(i,p\right)}$ between the LED *i* and the photodetector *p* can be expressed as:

(2) $$Hlos_{\left(i,p\right)}=\left\{\begin{array}{cc} \frac{A_{t}\left(m_{1}+1\right)}{2\pi d_{\left(i,p\right)}^{2}}cos^{m_{1}}\left(\phi \right)T_{s}\left(\Psi \right)g\left(\Psi \right)cos\left(\Psi \right), & 0\leq \Psi \leq \Psi_{c}\\ 0 & elsewhere \end{array}\right..$$

Here, $A_{t}$ is the physical area of the photodetector within the receiver, $d_{\left(i,p\right)}$ is the distance between the LED *i* and the photodetector *p*, $T_{s}\left(\Psi \right)$ is the gain of the optical filter, $\Psi_{c}$ denotes the width of the field of vision of the photodetector, and $m_{1}$ is the Lambertian emission order and is given by the semi-angle half-power LED as follows:

(3) $$m_{1}=\frac{-ln(2)}{ln(cos\theta_{\frac{1}{2}})}.$$

Furthermore, the gain of the optical concentrator g(Ψ) is given by the following expression:

(4) $$g\left(\Psi \right)=\left\{\begin{array}{cc} \frac{n^{2}}{sen^{2}\Psi_{c}}, & 0\leq \Psi \leq \Psi_{c}\\ 0 & \Psi >\Psi_{c} \end{array}\right.,$$

where *n* represents the refractive index.

Based on the three-dimensional coordinate system described in Figure 4, it is possible to find an expression for the irradiation angle as

(5) $$cos\left(\phi \right)=\frac{z_{i}-z_{p}}{{\left[{\left(z_{i}-z_{p}\right)}^{2}+{\left(x_{i}-x_{p}\right)}^{2}+{\left(y_{i}-y_{p}\right)}^{2}\right]}^{1/2}}$$

The model also assumes a control system located on each of the safety helmets, which uses an accelerometer to always keep the transceiver parallel to the roof of the mine, thereby cos(ϕ)=cos(Ψ). Therefore, the channel response of the system could be written as follows.

(6) $$Hlos_{\left(i,p\right)}=\frac{T_{s}\left(\Psi \right)g\left(\Psi \right)A_{t}\left(m_{1}+1\right){\left(z_{i}-z_{p}\right)}^{m_{1}+1}}{2\pi {\left[{\left(z_{i}-z_{p}\right)}^{2}+{\left(x_{i}-x_{p}\right)}^{2}+{\left(y_{i}-y_{p}\right)}^{2}\right]}^{\frac{m_{1}+3}{2}}}$$

Finally, the power received by the photodetector is given by:

(7) $$Pr_{\left(i,p\right)}=\frac{Pt_{i}T_{s}\left(\Psi \right)g\left(\Psi \right)A_{t}\left(m_{1}+1\right){\left(z_{i}-z_{p}\right)}^{m_{1}+1}}{2\pi {\left[{\left(z_{i}-z_{p}\right)}^{2}+{\left(x_{i}-x_{p}\right)}^{2}+{\left(y_{i}-y_{p}\right)}^{2}\right]}^{\frac{m_{1}+3}{2}}}$$

### 4.2. RF Uplink

**Figure 5:**
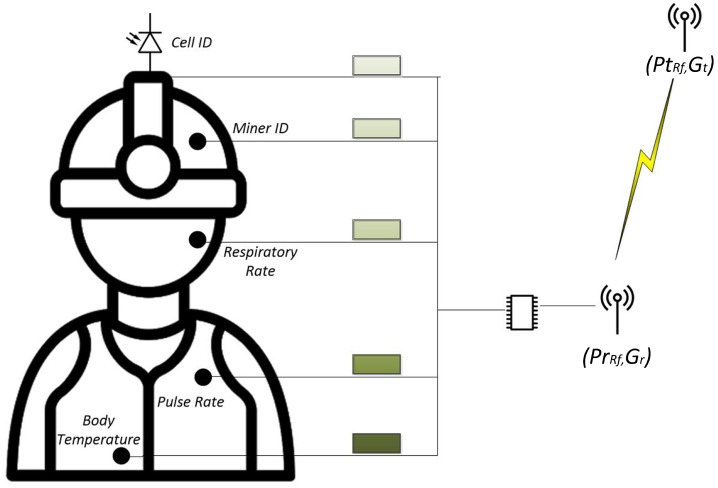
RF uplink.

In this work, a Wireless Body Area Network (WBAN) is proposed, which is a network that operates in the miner’s body, as shown in Figure 5. In this figure, the two RF antennas represent the transmitting and receiving antennas. The notation ($Pt_{RF}$, $G_{t}$) refers to the power transmitted and the gain of the transmitting antenna. In the receiving antenna, the notation ($Pr_{RF}$, $G_{r}$) represents the minimum power received and the gain of the receiving antenna. This notation is consistent with the Friis Transmission Equation used for the link budget calculation.

On the other hand, the WBAN network works with small batteries and has low transmission power, very low power consumption, typically below 10 mW, and low data throughput of around 10 Kbps. The proposed WBAN interconnects small nodes with vital sign monitoring devices; the three primary vital signs, body temperature, pulse rate, and respiratory rate, are taken by a wearable device attached to the miner. All of these monitoring devices, in turn, are connected to a central processor, which processes the information, including the Cell Identifier and the Miner’s Identifier, working in a specific format and converting the values to binary numbers and transmitting them using RF technology. These data are transferred through the infrastructure to a medical center where doctors evaluate the data received and assess the worker’s state of health. This WBAN enables the integration of automated monitoring results, diagnostics, alarms, emergencies, and electronic patient records databases for better patient health management.

**Figure 6:**
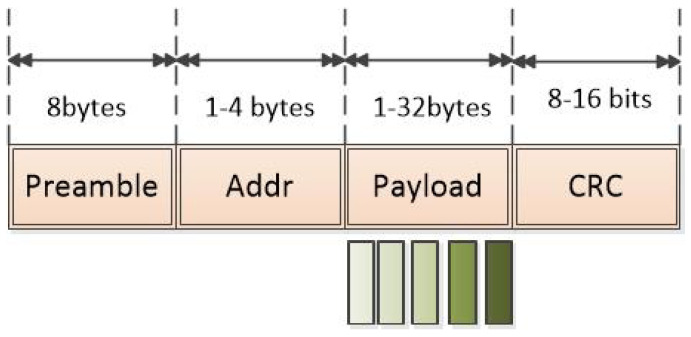
RF Frame.

To send the primary vital signs data, a single-chip radio transceiver for the 433/868/915 MHz ISM band is used. The current consumption is very low, in transmission only 11 mA at an output power of −10 dBm, and reception mode 12.5 mA. The module uses Gaussian frequency-shift keying (GFSK) modulation and Manchester encoding. The transmission mode is ShockBurst which transfers the frame as seen in Figure 6 [36].

The preamble is used to obtain the synchronization, an address field to establish communication, the payload field where the data of the acquired vital signs are sent, and a CRC field for error detection.

For a link budget, we use the Friis Transmission Equation that relates the power received $Pr_{RF}$ to the power transmitted $Pt_{RF}$ between two antennas separated by a distance *R*.

(8) $$Pr_{RF}=Pt_{RF}G_{t}G_{r}{\left(\frac{\lambda }{4\pi R}\right)}^{n},$$

where $G_{t}$ is the gain of the transmitting antenna, $G_{r}$ is the gain of the receiving antenna, and the term ${\left(\frac{\lambda }{4\pi R}\right)}^{2}$ is called the free-space loss factor where *n* can be modified depend of the environment.

## 5. Results and Discussion

The parameters used in the simulations are summarized in Table 2 and Table 3 the dimensions of the tunnel of (10 m, 5 m, 5 m) were obtained from [37]. The characteristics of both the photodetectors and the LEDs, in the visible and infrared range, were obtained from different technical sheets of commercial devices.

**Figure 7:**
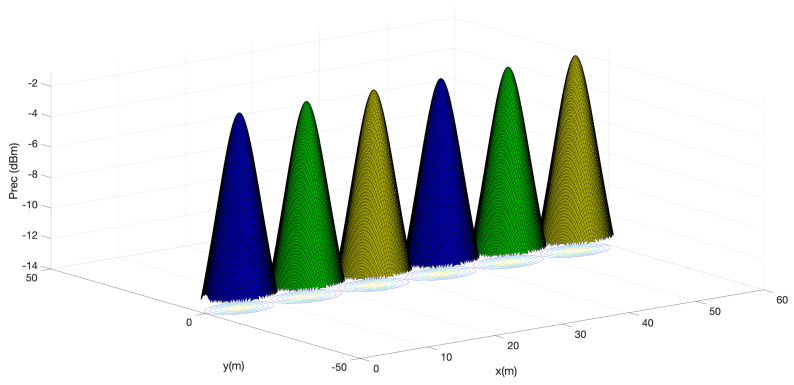
Optical power distribution for Cell ID LOS.

As shown in Figure 7, the performance of the proposed system in the downlink using the LEDs placed on the ceiling of the underground tunnel and the photodetector placed on the miner’s helmet is depicted to visualize the concept of proposed cells. Clearly, lobes that maximize power in the center of the cells and attenuate in the outer parts of the same can be distinguished. This result agrees with the expected behavior of the system, since the LED and photodetectors are sensitive to the presence of an object in the tunnel and their location in the scenario. This result is also in agreement with the results obtained in [37]. Furthermore, it is possible to observe that although the power received decreases in certain parts of the underground tunnel, it is never completely lost. Therefore, signal reception is ensured throughout the environment.

**Figure 8:**
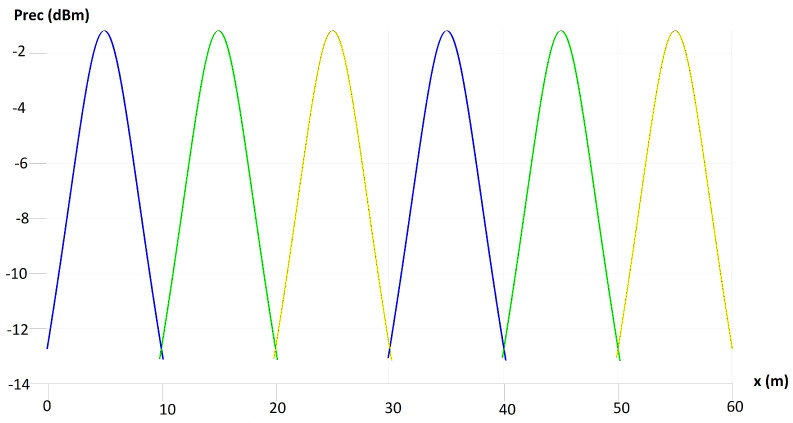
Profile of received power for VLC downlink LOS.

Figure 8 presents the profile of the power received for the VLC downlink LOS. The graph depicts the relationship between distance and received power, showing how received power changes as the distance between the LED transmitters and the photodetectors on the safety helmets of mining personnel varies. This information is crucial to understanding the signal strength and coverage of the VLC downlink communication system in the mining environment. The received power for the VLC downlink LOS is determined by various factors such as the physical area of the photodetector, the distance between the LED and the photodetector, the gain of the optical filter, the width of the field of vision of the photodetector, and the Lambertian emission order. Understanding this profile is essential to optimize the placement of LED transmitters and photodetectors to ensure reliable and efficient communication within the mining environment.

This analysis is important to evaluate the feasibility and effectiveness of the proposed hybrid architecture for VLC and RF communications in the context of mobile health monitoring of workers in the mining industry. By examining the profile of the power received for the VLC downlink LOS, researchers and industry professionals can make informed decisions regarding the implementation and deployment of the communication system.

Table 3 presents the simulation parameters for the RF uplink in the proposed hybrid communication system for mobile health monitoring in the mining industry. The RF system operates at a center frequency of 868 MHz, with a path loss exponent of 2.2. The RF transmitter has a transmitted power of 10 dBm and an antenna gain of 2.5 dBi. On the other hand, the RF receiver has a minimum power received (sensitivity) of −100 dBm and an antenna gain of 2.5 dBi. These parameters are essential to understand the performance and characteristics of RF uplink communication in the proposed system.

**Figure 9:**
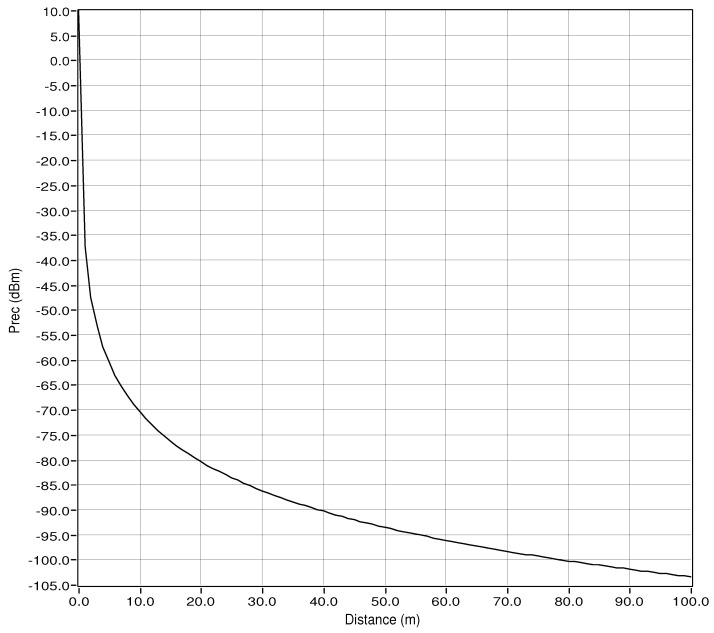
Received power for RF uplink.

The power received for the RF uplink, as shown in Figure 9. This curve shows a gradual decrease in power received as the distance between the RF transmitter and the RF receiver increases. This behavior is in line with the expected path loss characteristics in wireless communication systems, where the received power diminishes as the distance between the transmitter and receiver increases.

The causes of this gradual decrease in received power can be attributed to factors such as free-space loss, antenna gains, and the path loss exponent. Free-space loss, which is influenced by the distance between the transmitter and receiver, results in a reduction of signal strength as the distance increases. Furthermore, antenna gains of transmitting and receiving antennas play a role in the received power, as they affect the effective radiated power and the ability to capture the transmitted signal. The path loss exponent, which accounts for the environment’s impact on signal propagation, also contributes to the observed power-distance relationship.

Finally, environmental factors such as obstacles, reflections, and absorption of the RF signal in the mining environment can also influence the received power. These factors can cause signal attenuation and contribute to a gradual decrease in received power as the distance increases.

## 6. Conclusions

The proposed hybrid architecture for VLC and RF communications, based on IIoT, presents a promising solution to monitor the mobile health of workers in the mining industry. The use of VLC for downlink positioning and RF communication for uplink vital signs data transmission demonstrates the feasibility of this architecture. The results of the coverage and transmission analysis indicate that the proposed system effectively addresses the challenges of mobile health monitoring in mining environments. The findings support the viability of the hybrid architecture, providing valuable information for the implementation and deployment of the communication system. This research contributes to the advancement of m-Health technologies and underscores the potential of the proposed hybrid communication system to improve worker safety and health in the mining industry. Future work could focus on further optimizing the system’s performance, considering factors such as environmental influences and potential integration with advanced data analytics for real-time health monitoring and predictive maintenance in mining operations.

## Figures and Tables

**Table 1:** Comparison of wireless communication technologies.

Property	VLC	IR	Bluetooth	Wi-Fi	Zigbee
Bandwidth	Unlimited, 400–700 nm	Unlimited, 800–1600 nm	Regulated and limited	Regulated and limited	Regulated and limited
Line of sight	Yes	Yes	No	No	No
Standards	IEEE 802.15.7 [13]	IrDa	IEEE 802.15.1 [6]	IEEE 802.11 [8]	IEEE 802.15.4 [10]
Power consumption	Relatively Low	Relatively low	Relatively low	Medium	Relatively low
Coverage	Limited	Limited	Good	Good	Good

**Table 2:** Simulation parameters for VLC downlink.

Component	Description	Variable	Value
VLC Transmitter	Power transmitted	$Pt_{i}$	10 W
	Half Power Angle	$cos\theta_{\frac{1}{2}}$	60°
VLC Receiver	Physical surface area	$A_{t}$	1 cm${}^{2}$
	Gain of the optical filter	$T_{s}\left(\Psi \right)$	1
	Refractive Index	*n*	1.5
	Field of View	$\Psi_{c}$	60°

**Table 3:** Simulation parameters for RF uplink.

Component	Description	Variable	Value
RF system	Center Frequency	*f*	868 MHz
	Path loss exponent	*n*	2.2
RF Transmitter	Power transmitted	$Pt_{RF}$	10 dBm
	Antenna gain	$G_{t}$	2.5 dBi
RF Receiver	Minimum power received (Sensitivity)	$Pr_{RF}$	−100 dBm
	Antenna gain	$G_{r}$	2.5 dBi

## Data Availability

Data are contained within the article.

## Abbreviations

The following abbreviations are used in this manuscript:

ECG	Electrocardiogram
EEG	Electroencephalogram
HB-LED	High Brightness Light Emitting Diode
ILO	International Labour Organization
IR	Infra Red
LED	Light Emitting Diode
LOS	Line Of Sight
MINER	Mine Improvement and New Emergency Response
OBAN	Optical Body Area Network
OFDM	Orthogonal Frequency Division Multiplexing
OOK	On-Off Keying
OWC	Optical Wireless Communication
PLC	Power Line Communication
PPG	Photoplethysmography
RGB	Red, Green and Blue
TH	Time-Hopping
SCMA	Sparse Code Multiple Acces
TDMA	Time Division Multiple Access
VLC	Visible Light Communications
WBAN	Wireless Body Area Network
